# Prognostic significance of immunohistochemical classification utilizing biopsy specimens in patients with extensive-disease small-cell lung cancer treated with first-line chemotherapy and immune checkpoint inhibitors

**DOI:** 10.1007/s00432-024-05652-2

**Published:** 2024-03-14

**Authors:** Naoki Shijubou, Toshiyuki Sumi, Terufumi Kubo, Kenta Sasaki, Tomohide Tsukahara, Takayuki Kanaseki, Kenji Murata, Yoshiko Keira, Kotomi Terai, Tatsuru Ikeda, Yuichi Yamada, Hirofumi Chiba, Yoshihiko Hirohashi, Toshihiko Torigoe

**Affiliations:** 1 Department of Pathology, Sapporo Medical University School of Medicine, Sapporo, 060-8556 Japan; 2 Department of Respiratory Medicine and Allergology, Sapporo Medical University School of Medicine, Sapporo, Hokkaido Japan; 3 Department of Respiratory Medicine, Hakodate Goryoukaku Hospital, Hakodate, Hokkaido Japan; 4 Department of Pathology, Hakodate Goryoukaku Hospital, Hakodate, Hokkaido Japan

**Keywords:** Extensive-disease small-cell lung cancer, CD8-positive cell, HLA-class I, Chemoimmunotherapy, Real clinical setting

## Abstract

**Purpose:**

Although immune checkpoint inhibitors (ICIs), together with cytotoxic chemotherapy (chemoimmunotherapy), have been adapted for the initial treatment of extensive-disease small-cell lung cancer (ED-SCLC), they have achieved limited success. In ED-SCLC, a subtype of SCLC, the expression of immune-related molecules and clinical data are not well understood in relation to ICI treatment efficiency.

**Methods:**

We examined lung biopsy specimens from patients diagnosed with ED-SCLC treated with chemoimmunotherapy or chemotherapy. SCLC subtype, expression of HLA class I, and infiltration of CD8-positive cells were examined using immunohistochemistry (IHC). Subsequently, the association between clinical factors, IHC results, and progression-free survival or overall survival was assessed.

**Results:**

Most of the cases showed the achaete-scute homolog 1 (ASCL1) subtype. Among the 75 SCLC cases, 29 expressed high levels of HLA class I, while 46 showed low levels or a negative result; 33 patients were characterized as CD8-high, whereas 42 were CD8-low. In the chemoimmunotherapy cohort, multivariate analysis revealed a correlation between CD8-high and improved survival. Specifically, patients in the CD8-high group of the chemoimmunotherapy cohort experienced enhanced survival compared to those in the chemotherapy cohort, which was attributed to ICI addition. IHC subtype analysis demonstrated a survival advantage in the SCLC-I and SCLC-A groups when ICI was combined with chemotherapy compared to chemotherapy alone.

**Conclusion:**

Our study highlights the predictive value of IHC-classified subtypes and CD8-positive cell infiltration in estimating outcomes for patients with ED-SCLC treated with chemoimmunotherapy as a first-line therapy. These findings have practical implications for daily clinical assessments and treatment decisions.

**Supplementary Information:**

The online version contains supplementary material available at 10.1007/s00432-024-05652-2.

## Introduction

Small-cell lung cancer (SCLC) is the third most common histological subtype of lung cancer, accounting for approximately 15% of all lung cancer cases. SCLC has a poor prognosis because it is often identified at an advanced stage and SCLC cells are highly malignant. While early-stage SCLC has a 15–30% 5-year survival rate, less than 1% of patients with extensive disease (ED) survive for more than 5 years (Gazdar et al. 2017; Byers and Rudin 2015). In the past decade, the success of immune checkpoint inhibitors (ICIs) in the treatment of various types of malignancies has inspired a paradigm shift in cancer treatment (Herbst et al. 2016). Beyond ICI monotherapy, the combined use of chemotherapeutic agents with ICIs (chemoimmunotherapy) is widely reported. However, this combination therapy has shown limited improvements in the survival of patients with ED-SCLC (Paz-Ares et al. 2019; Horn et al. 2018). Therefore, developing a clinically adequate and feasible strategy to identify patients who could benefit from chemoimmunotherapy is necessary.

Although SCLC has long been regarded as a unique histological entity, its modern classification is based on the expression of several transcription-related factors (Zhang et al. 2018; Carney et al. 1985). The four molecular subtypes of SCLC include SCLC-A, SCLC-N, SCLC-P, and SCLC-Y, which express achaete-scute homolog 1 (ASCL1), neurogenic differentiation factor 1 (NEUROD1), POU class 2 homeobox 3 (POU2F3), and yes-associated protein 1 (YAP1), respectively. SCLC-A and SCLC-N, which show neuroendocrine differentiation, constitute the major populations (Zhang et al. 2018; Rudin et al. 2019), whereas SCLC-P and SCLC-Y, which lack neuroendocrine characteristics, are rare. This classification is based on gene expression; however, several investigations using immunohistochemistry (IHC) were inconsistent in the verification of the SCLC-Y subtype, due to the low protein expression of YAP1 in SCLC-Y cells (Baine et al. 2020). Alternatively, SCLC with low expression of ASCL1, NEUROD1, and POU2F3 in IHC have been classified as SCLC-I owing to their high expression of immune-related genes (Gay et al. 2021).

While the IMpower133 trial reported a significant survival benefit for the SCLC-I subtype employing retrospective transcriptome analysis, the effect of routine first-line chemoimmunotherapy treatment on the various histopathological phenotypes of SCLC, such as the subtype of SCLC or patient immunological characteristics, is unclear (Gay et al. 2021). In this study, we investigated the relationship between IHC-classified subtypes, HLA class I expression in tumor cells, CD8-positive cell infiltration into the tumor parenchyma, clinical data, progression-free survival (PFS), and overall survival (OS). Furthermore, a comparative analysis was conducted between the chemoimmunotherapy group and the chemotherapy group to assess the efficacy of ICIs. Notably, this study utilized formalin-fixed paraffin-embedded (FFPE) bronchoscopic biopsy specimens from patients diagnosed with ED-SCLC and treated with chemoimmunotherapy as the initial therapy in a real clinical setting.

## Materials and methods

### Patients and clinical data

We retrospectively analyzed the clinical data and biopsy specimens of 42 patients diagnosed with ED-SCLC who started chemoimmunotherapy as first-line treatment between August 1, 2018, and December 31, 2022, at Hakodate Goryokaku Hospital (Hokkaido, Japan). As a chemotherapy cohort, a retrospective analysis of 39 patients who received first-line chemotherapy between January 2017 and August 2018, prior to approval of chemoimmunotherapy for ED-SCLC in Japan, was also performed in the same manner. Informed consent was obtained from all patients as opt-out forms on the hospital website. The study protocol was designed in accordance with the guidelines of the Declaration of Helsinki and approved by the Institutional Review Board of Hakodate Goryokaku Hospital (permit number 2023-005).

Use of chemoimmunotherapy or chemotherapy as the first-line therapy was selected based on the following criteria: Eastern Cooperative Oncology Group performance status (ECOG-PS) 0–2, surgically unresectable and not amenable to radical chemoradiotherapy, and stage IV or stage III ED-SCLC or postoperative recurrence that was not surgically resectable and not amenable to radical chemoradiotherapy. Data on age, sex, best response, PS, and treatment-related adverse events were collected retrospectively from medical records, and PS was classified as 0–4 using the ECOG index.

### IHC analysis and evaluation of tissue staining

Tumor tissues were fixed in 10% buffered formalin and embedded in paraffin. Sections (4 µm thick) of FFPE tumor tissues were stained with the following monoclonal antibodies after epitope retrieval using Target Retrieval Solution, pH 9 (DAKO, Glostrup, Denmark). To classify SCLC subtypes, mouse anti-MASH1 (ASCL1) monoclonal antibody (clone 24B72D11.1; eBioscience, San Diego, CA, USA), rabbit anti-NEUROD1 monoclonal antibody (clone EPR20766, Abcam, Cambridge, UK), and mouse anti-POU2F3 (clone 6D1, Santa Cruz Biotechnology, Dallas, TX, USA) were used. To detect CD8 and HLA class I molecules, mouse anti-CD8 monoclonal antibody (clone C8/144B, DAKO) and mouse anti-HLA-A, -B, and -Cw monoclonal antibodies (clone EMR8-5; Hokudo, Sapporo, Japan) were used, respectively. Antibodies were used according to the manufacturer’s instructions.

We classified SCLC cases into four subtypes: SCLC-A, SCLC-N, SCLC-P, and SCLC-I. Subtypes of SCLC were determined by the percentage of positive cells for each of ASCL1, NEUROD1, and POU2F3. According to their expression profiles, ASCL1-, NEUROD1-, and POU2F3-dominant cases were designated as SCLC-A, SCLC-N, and SCLC-P, respectively. If SCLC did not express ASCL1, NEUROD1, or POU2F3 in more than 95% of cases, the subtype was defined as SCLC-I.

The expression of HLA class I molecules was divided into three patterns: A high expression pattern corresponded to the expression of HLA class I molecules on the membrane of tumor cells comparable to that of the vascular endothelium in the surrounding stroma. Low expression patterns corresponded to a low expression of HLA class I molecules in tumor cells. Negative patterns indicated the complete absence of HLA class I molecule expression in tumor cells. CD8-high group was defined as > 10 CD8 expressing cells in the tumor per high-power field (0.24 mm^2^), while the remaining cases were designated as CD8-low. To obtain concordant IHC results, each slide was examined and discussed by two pathologists and a pulmonologist using a multiheaded microscope.

### Statistical analysis

PFS was defined as the time from the date of the first treatment to the date of progressive disease (PD) determination or last follow-up. OS was defined as the time from the date of the first treatment to the date of death or last follow-up. Survival was evaluated from the date of the first treatment until July 31, 2023. All variables are presented as medians and 95% confidence intervals (CIs) or medians and ranges. Fisher’s exact test was used to determine the association between each factor. Cox proportional hazards regression models were performed as univariate analyses for each factor, and all significant factors (*P* < 0.10) were combined. The significance level for analyses other than univariate analysis of the Cox proportional hazards regression model was 5%. Survival curves were generated using the Kaplan–Meier method for factors that were significant in the Cox proportional hazards regression model. All statistical analyses were performed using EZR (Saitama Medical Center, Jichi Medical University, Saitama, Japan), a graphical user interface for R (The R Foundation for Statistical Computing, Vienna, Austria); more specifically, it is a modified version of the R commander designed to add statistical functions that are frequently used in biostatistics (Kanda 2013).

## Results

### Classification of SCLC subtype and evaluation of HLA class I expression and CD8-positive cell infiltration

**Fig. 1:**
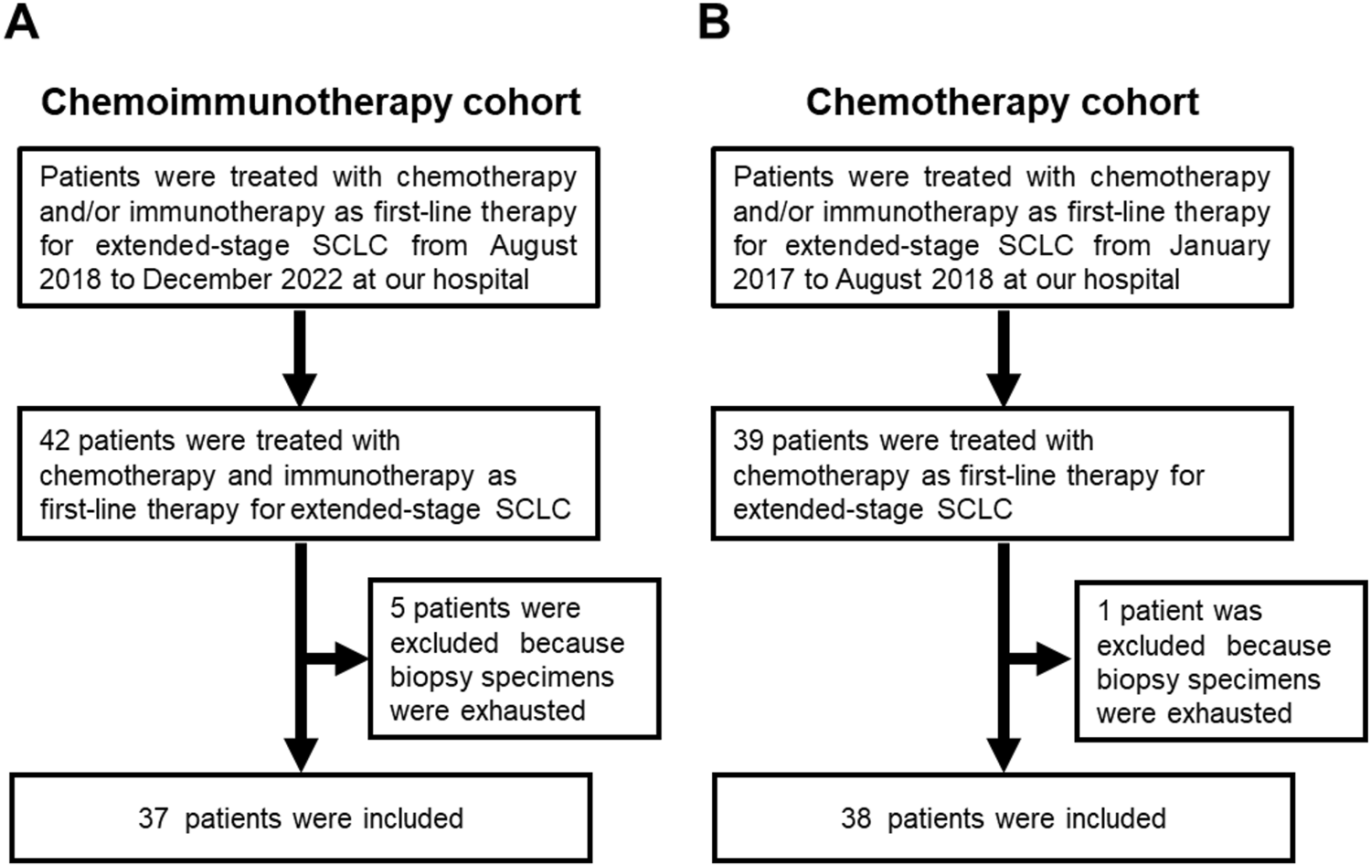
Study workflow. **A** Chemoimmunotherapy cohort. **B** Chemotherapy cohort

**Fig. 2:**
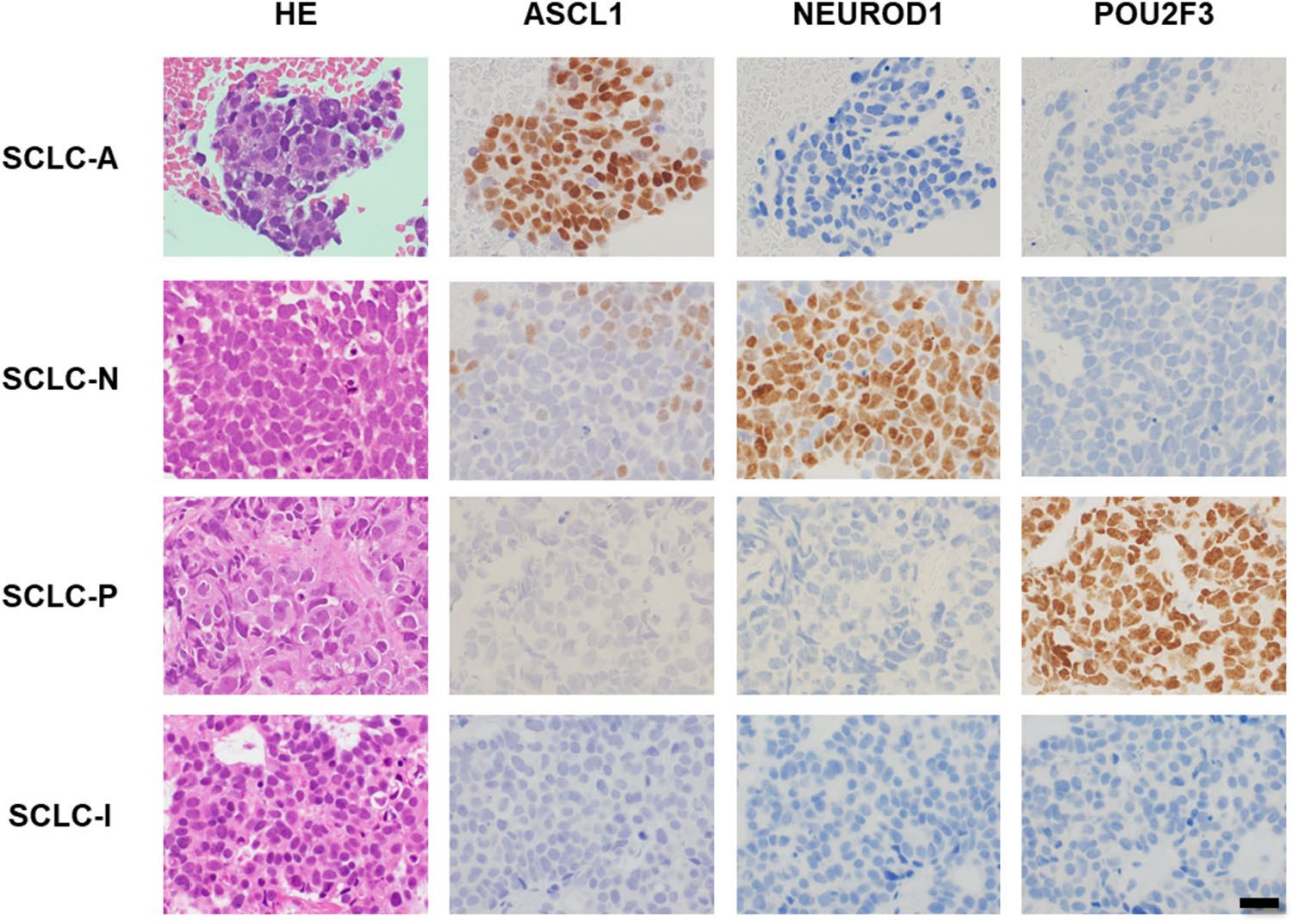
Classification of SCLC according to IHC of ASCL1, NEUROD1, and POU2F3

Among the 42 patients who received chemoimmunotherapy as first-line treatment for ED-SCLC, five were excluded from evaluation because of the exhaustion of FFPE specimens (Fig. 1A). In the same manner, among the 39 patients who received chemotherapy as first-line treatment for ED-SCLC, a single case was excluded from the investigation (Fig. 1B). The clinical characteristics of the included patients are shown in Table 1. We classified the included SCLC cases based on the expression of ASCL1, NEUROD1, and POU2F3 using IHC. Representative IHC images for each classification are shown in Fig. 2. In the 37 and 24 cases of the chemoimmunotherapy and chemotherapy cohorts, 25 (67.6%), 5 (13.5%), 2 (5.4%), and 5 (13.5%), and, 24 (63.2%), 4 (10.5%), 4 (10.5%) and 6 (15.8%) were classified as SCLC-A, SCLC-N, SCLC-P, and SCLC-I, respectively.

**Table 1:** Baseline patient characteristics

Parameters	Chemoimmunotherapy cohort (*N* = 37)	Chemotherapy cohort (*N* = 38)
Sex N (% of total)		
Male	29 (78.9%)	32 (84.2%)
Female	8 (21.1%)	6 (18.8%)
Age		
Median (range)	67 (51–81)	70.5 (55–81)
Clinical stage (n [%])		
III	4 (10.8%)	5 (10.8%)
IVA	6 (16.2%)	5 (10.8%)
IVB	27 (73.0%)	28 (10.8%)
ECOG performance status N (% of total)		
0	26 (70.3%)	14 (36.8%)
≥ 1	11 (29.7%)	24 (63.2%)
SCLC Subtype N (% of total)		
SCLC-A	25 (59.1%)	24 (63.2%)
SCLC-N	5 (18.2%)	4 (10.5%)
SCLC-P	2 (9.1%)	4 (10.5%)
SCLC-I	5 (13.6%)	6 (15.8%)
HLA-class I N (% of total)		
Negative	4 (6.5%)	3 (7.9%)
Low	17 (45.9%)	22 (57.9%)
High	16 (43.2%)	13 (34.2%)
CD8 N (% of total)		
Positive	17 (45.9%)	16 (42.1%)
Negative	20 (54.1%)	22 (57.9%)
irAE (grade ≥ 3) N (% of total)	3 (8.1%)	NA
Best response N (% of total)		
PD	4 (10.8%)	12 (31.6%)
SD	5 (13.5%)	5 (13.2%)
PR	26 (70.3%)	21 (55.3%)
CR	2 (5.4%)	0 (0.0%)

*CR* complete response, *irAE* immune-related adverse event, *NA* not applicable, *PD* progressive disease, *PR* partial response, *SD* stable disease

Representative images of SCLC sub-classified by ASCL1, NEUROD1, and POU2F3 expression. Dominant nuclear expression of ASCL1, NEUROD1, and POU2F3 in SCLC corresponded with SCLC-A, SCLC-N, and SCLC-P, respectively. If ASCL1, NEUROD1, or POU2F3 were not expressed in more than 95% of cases, the subtype was designated as SCLC-I. Bar = 20 μm. Original magnification:  400×.

**Fig. 3:**
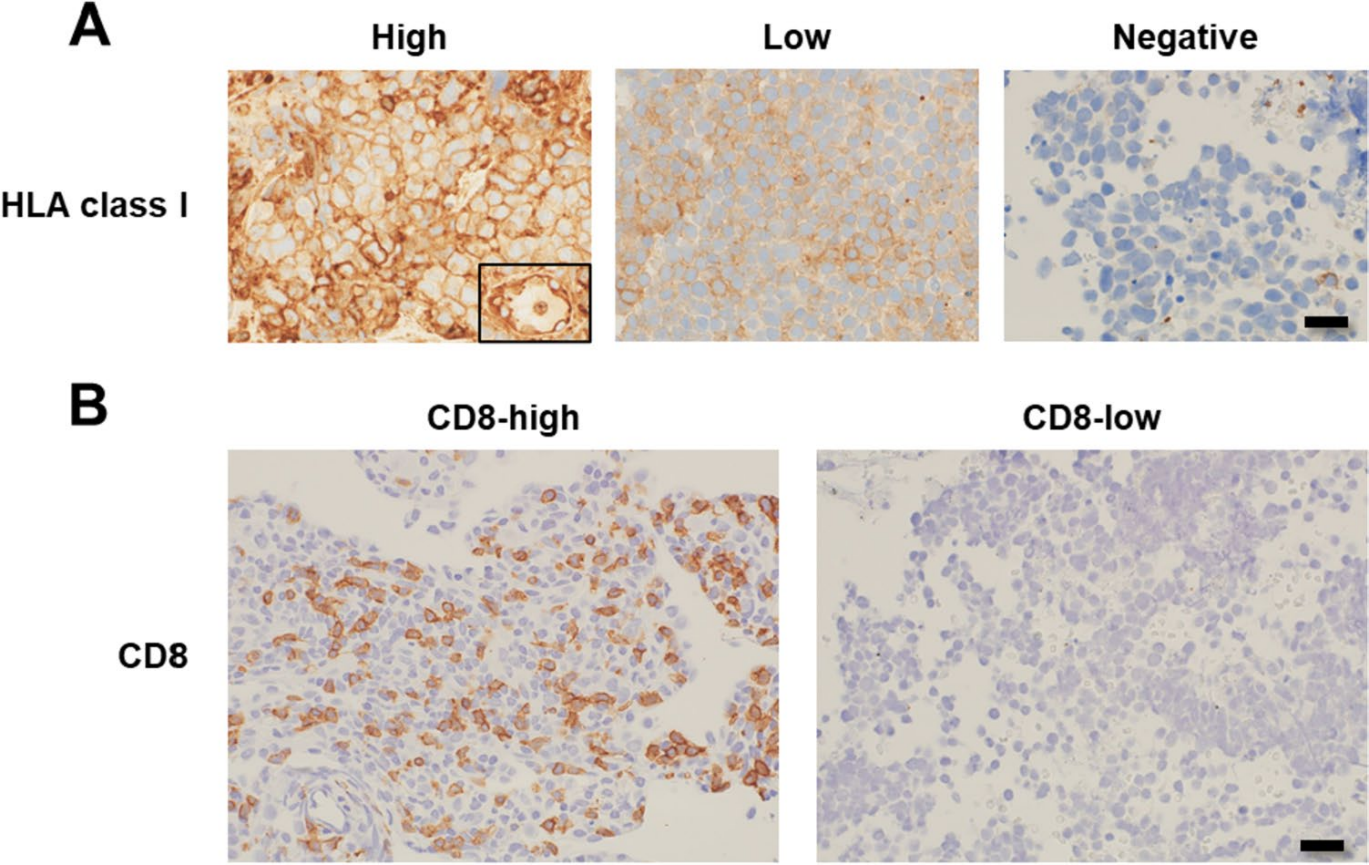
IHC of immune-related molecules. **A** Three patterns of HLA class I molecule expression. High: the expression of HLA class I molecules on the membrane of tumor cells is comparable to that on the vascular endothelium in the surrounding stroma (inset). Low: HLA class I molecules are faintly expressed in the tumor cells. Negative: complete absence of HLA class I molecule expression in the tumor cells. Bar = 20 μm. Original magnification: × 400. **B** Positive and negative correspond to more and less than 10 CD8-positive cells per high power field (0.24 mm^2^), respectively. Bar = 20 μm. Original magnification: × 400

We investigated the expression of HLA class I molecules, a putative prerequisite for ICI-mediated cancer immunotherapy, in SCLC cells (Fig. 3A). While 16 (43.2% of chemoimmunotherapy) and 13 (34.2% of chemotherapy) patients expressed high levels of HLA class I molecules, 17 (45.9% of chemoimmunotherapy) and 22 (57.9% of chemotherapy) or four (10.8% of chemoimmunotherapy) and three (7.9% of chemotherapy) showed low or negative HLA class I expression, respectively. Next, we investigated the infiltration of cytotoxic T lymphocytes (CTLs) as a surrogate marker for the antitumor reaction by evaluating the infiltration of CD8 expressing cells into the tumor (Fig. 3B). Seventeen (45.9% of chemoimmunotherapy) and 17 (44.7% of chemotherapy) cases were CD8-high, and 20 (54.1% of chemoimmunotherapy) and 21 (55.3% of chemotherapy) were CD8-low. Notably, 9/11 (81.8% of total SCLC-I) cases were classified as CD8-high. Fisher’s exact test indicated the statistically significant association between CD8 and SCLC-I (*P* = 0.0085), while the other type did not show such an association.

### Prognostic implication of the SCLC subtype, tumor HLA class I expression, and CD8-positive cell infiltration in chemoimmunotherapy

**Fig. 4:**
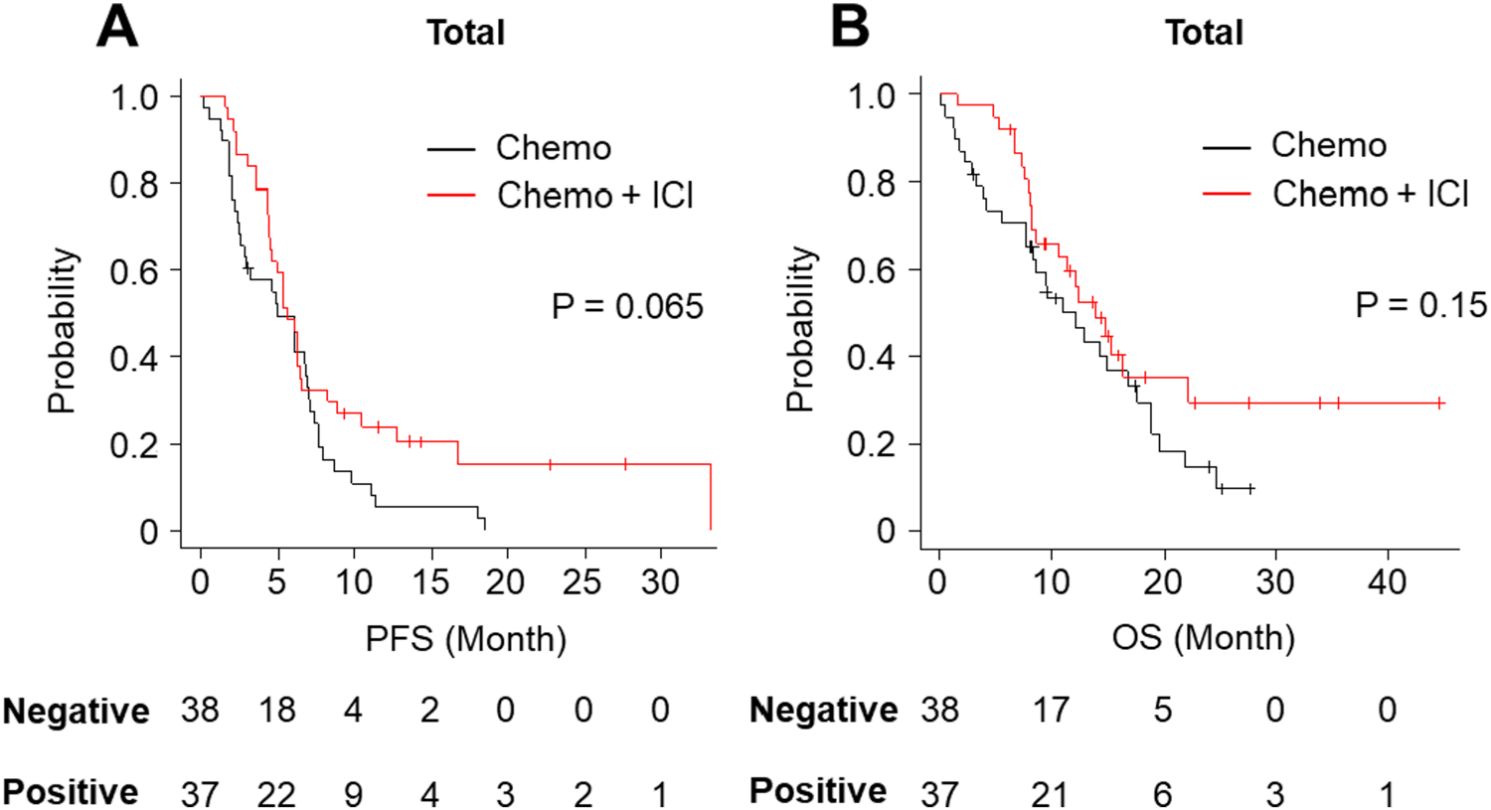
Kaplan–Meier estimates of PFS and OS in total. **A** PFS of chemoimmunotherapy cohorts and chemotherapy cohorts. **B** OS of chemoimmunotherapy cohort (chemo + ICI) and chemotherapy cohort (chemo)

The median PFS of the initial therapy of the chemoimmunotherapy cohort and that of the chemotherapy cohort were 5.7 months (95% CI 4.5–6.6 months) and 4.9 months (95% CI 2.5–6.9 months), respectively. On the other hand, the median OS of the initial therapy of the abovementioned cohorts were 13.9 months (95% CI 8.6–22.2 months) and 12.1 months (95% CI 7.7–16.8 months), respectively. Kaplan–Meier survival analysis indicated that ICI combination had a slightly favorable trend with PFS (*P* = 0.065) and OS (*P* = 0.15) (Fig. 4A, B). Notably, seven patients (18.9%) in the chemoimmunotherapy group survived following the initial treatment at the time of data cutoff. Among them, the longest observed survival was 45 months. No significant associations in either group were found between age, sex, clinical stage, PS, treatment-related adverse events, subtype, HLA class I expression, or infiltration of CD8-positive cells, as evaluated by Fisher’s exact test.

Cox regression analysis was performed for the SCLC subtype, tumor HLA class I expression, and CD8-positive cell infiltration, in addition to age, sex, clinical stage, and PS. In the univariate analysis in the chemoimmunotherapy cohort, CD8-high (*P* = 0.0060) showed a significant association with PFS, while ECOG-PS ≥ 1 (*P* = 0.0014) and SCLC-P (vs SCLC-I) (*P* = 0.037) had an unfavorable association, and clinical stage (C-Stage ≥ IVB) (*P* = 0.070) and SCLC-N (vs SCLC-I) (*P* = 0.068) showed an unfavorable trend with PFS (Table 2). Multivariate analysis of the parameters in the chemoimmunotherapy cohort significantly associated with PFS showed that CD8-high (*P* = 0.0061) was independently associated with favorable PFS. In contrast, ECOG-PS ≥ 1 (*P* = 0.028), SCLC-P (vs SCLC-I) (*P* = 0.037), and clinical stage (C-Stage ≥ IVB) (*P* = 0.043) were negatively correlated with prolonged PFS (Table 2). Univariate analysis in the chemoimmunotherapy cohort revealed that CD8-high (*P* = 0.0011) and ECOG-PS ≥ 1 (*P* = 0.0077) were significantly correlated with favorable OS. Multivariate analysis of the parameters in the chemoimmunotherapy cohort significantly associated with OS showed that CD8-high (*P* = 0.0018) was independently associated with favorable OS. In contrast, ECOG-PS ≥ 1 (*P* = 0.024) was negatively correlated with prolonged OS (Table 3).

**Table 2:** Univariate and multivariate analysis of predictors of PFS in the chemoimmunotherapy cohort

Parameters	Univariate analysis HR (95% CI)	*P*-value	Multivariate analysis HR (95% CI)	*P*-value
Age ≥ 70 years	1.04 (0.50–2.14)	0.92		
Sex, female	1.46 (0.59–3.62)	0.41		
ECOG-PS ≥ 1	3.73 (1.66–8.38)	0.0014	2.80 (1.12–6.99)	0.028
HLA I high	0.97 (0.47–2.00)	0.94		
CD8-high	0.34 (0.16–0.73)	0.0060	0.29 (0.12–0.70)	0.0061
Subtype SCLC-N (vs SCLC-I)	4.14 (0.90–19.07)	0.068	3.13 (0.63–15.57)	0.16
Subtype SCLC-P (vs SCLC-I)	7.41 (1.17–47.02)	0.037	10.77 (1.15–101.00)	0.037
Subtype SCLC-A (vs SCLC-I)	2.53 (0.75–8.60)	0.14	1.53 (0.43–5.36)	0.51
C-Stage ≥ IVB	2.30 (0.93–5.64)	0.070	2.93 (1.03–8.32)	0.043
Presence of severe irAE	0.70 (0.21–2.34)	0.57		

*CI* confidence interval, *HR* hazard ratio

**Table 3:** Univariate and multivariate analysis of predictors of OS in the chemoimmunotherapy cohort

Parameters	Univariate analysis HR (95% CI)	*P*-value	Multivariate analysis HR (95% CI)	*P*-value
Age ≥ 70 years	0.69 (0.28–1.67)	0.41		
Sex, female	1.24 (0.42–3.71)	0.69		
ECOG-PS ≥ 1	3.52 (1.39–8.80)	0.0077	3.00 (1.16–7.78)	0.024
HLA I high	0.74 (0.30–1.79)	0.50		
CD8-high	0.12 (0.034–0.43)	0.0011	0.13 (0.037–0.47)	0.0018
Subtype SCLC-N (vs SCLC-I)	NA	NA		
Subtype SCLC-P (vs SCLC-I)	NA	NA		
Subtype SCLC-A (vs SCLC-I)	NA	NA		
C-Stage ≥ IVB	1.40 (0.51–3.82)	0.51		
Presence of severe irAE	0.70 (0.16–3.08)	0.64		

*CI* confidence interval, *HR* hazard ratio, *NA* not applicable

In the univariate analysis in the chemotherapy cohort, ECOG-PS ≥ 1 (*P* = 0.010) had an unfavorable association and clinical stage (C-Stage ≥ IVB) (*P* = 0.16) had an unfavorable trend with PFS (Table S1). Multivariate analysis of the parameters in the chemotherapy cohort significantly associated with PFS showed that ECOG-PS ≥ 1 (*P* = 0.022) were negatively correlated with prolonged PFS (Table S1). Univariate analysis in the chemotherapy cohort revealed that ECOG-PS ≥ 1 had an unfavorable trend with OS (*P* = 0.058) (Table S2).

**Fig. 5:**
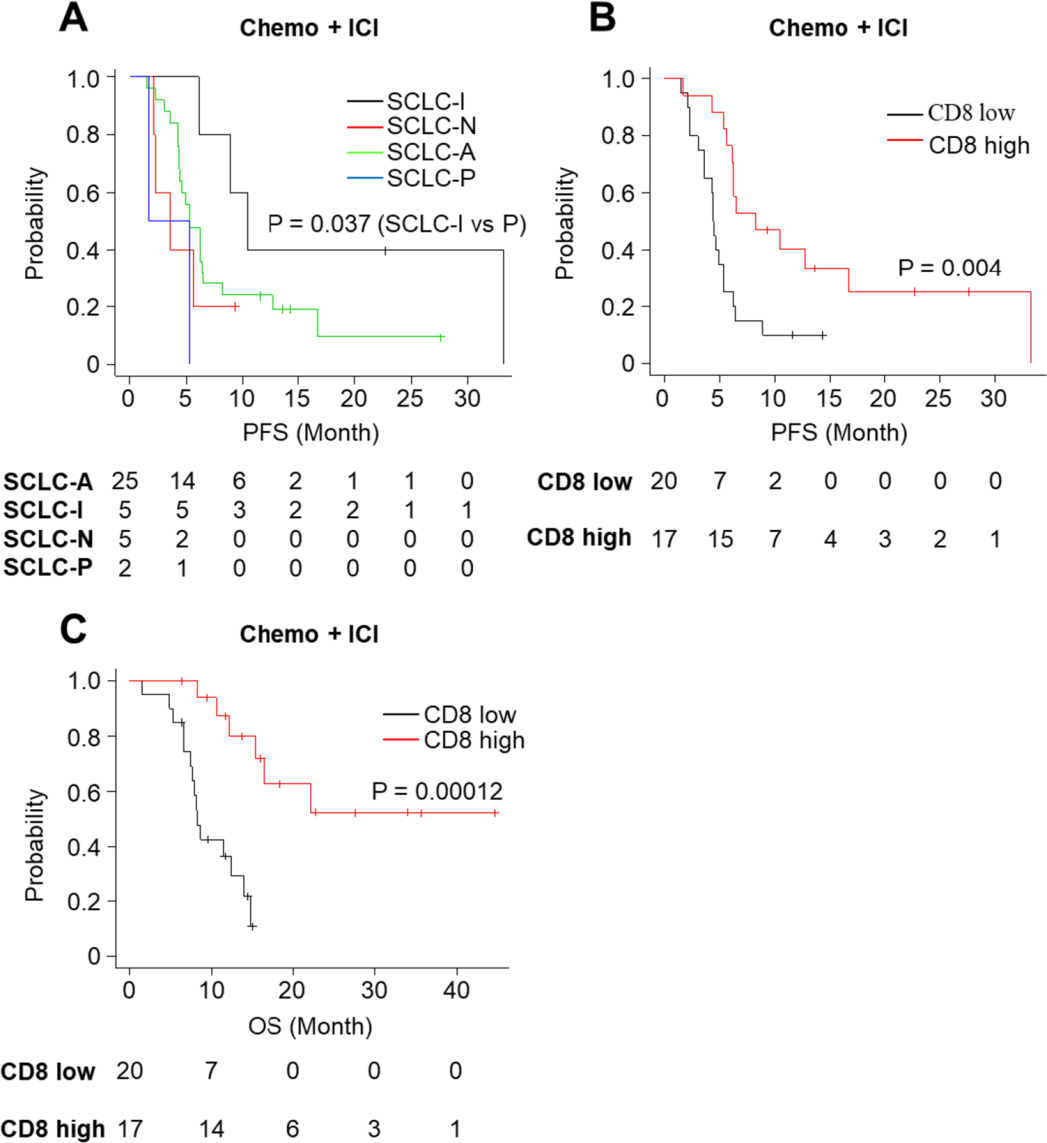
Kaplan–Meier estimates of PFS and OS in the chemoimmunotherapy (chemo + ICI) cohort. PFS classification based on the **A** SCLC subtype and **B** CD8-high or -low groups. **C** OS classification based on the CD8-high or -low groups

We conducted Kaplan–Meier survival analysis for tumor IHC-subtypes and CD8, revealing significant differences in PFS and overall OS in the chemoimmunotherapy cohort, as indicated by Cox regression analysis. Similar to the analysis with the Cox regression model, the prognosis of SCLC-P was found to be worse than that of SCLC-I (*P* = 0.037), although there were no significant differences in other group comparisons (Fig. 5A). In addition, CD8-high was associated with prolonged PFS (*P* = 0.004) and OS (*P* = 0.00012) (Fig. 5B, C, respectively).

### CD8-high was a good prognostic factor for ICI combination and ICI combination dramatically improved prognosis of SCLC-I

**Fig. 6:**
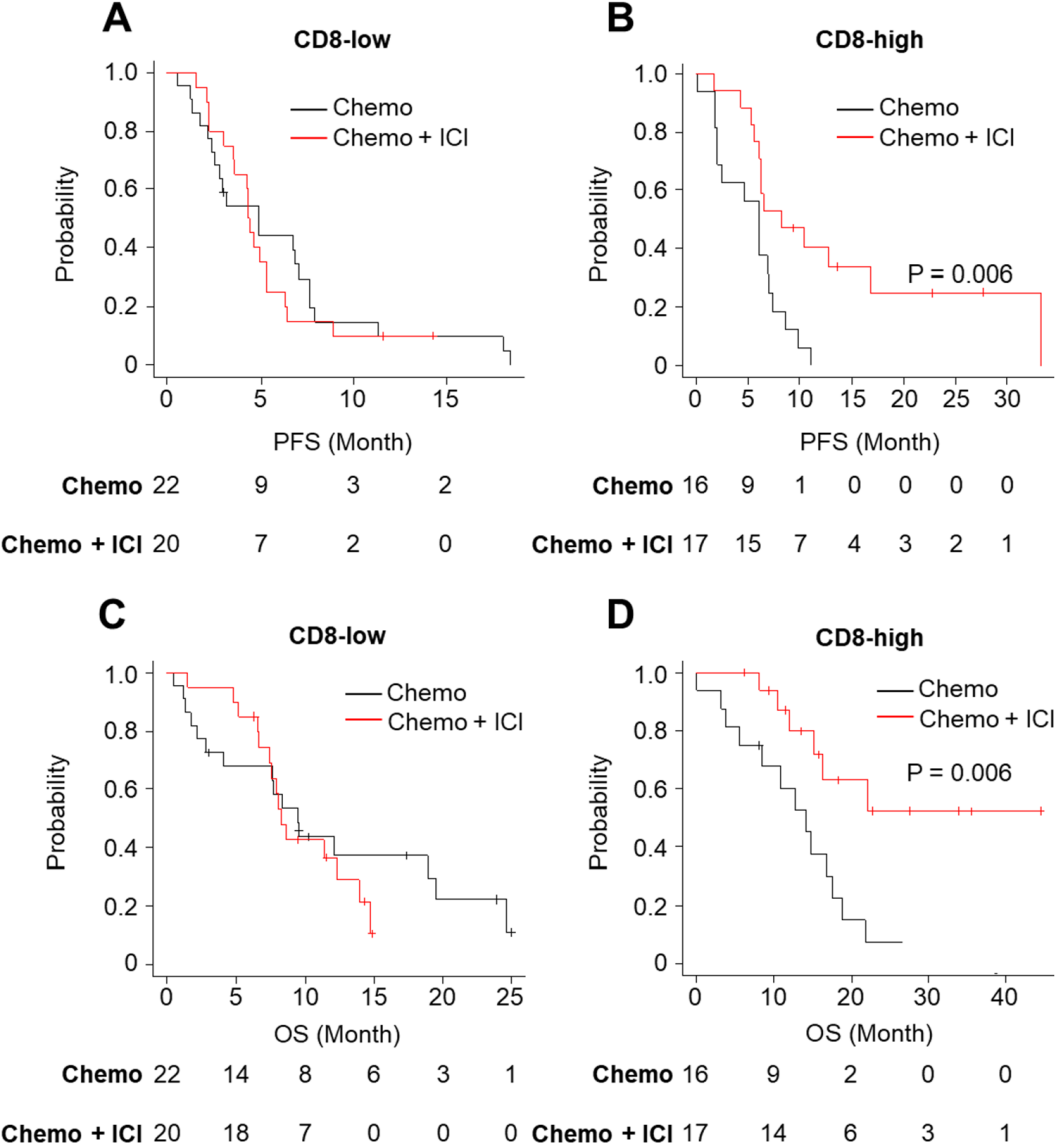
Kaplan–Meier estimates of PFS and OS for comparison of the chemoimmunotherapy (chemo + ICI) and the chemotherapy (chemo) cohorts. **A** PFS in the CD8-low group; **B** PFS in the CD8-high group; **C** OS in the CD8-low group; **D** OS in the CD8-high group

When comparing the chemoimmunotherapy cohort with the chemotherapy cohort, ICI addition was associated with a prolonged PFS (*P* = 0.006) (Fig. 6B) and OS (*P* = 0.006) (Fig. 6D) in the CD8-high group but not CD8-low group (Fig. 6A, C).

When further investigated based on IHC-classified subtypes, ICI combination dramatically improved PFS (*P* = 0.021) and OS (*P* = 0.0025) of SCLC-I (Fig. S1A and SB). However, ICI did not confer prolonged survival in other subtypes of SCLC (data not shown). In SCLC-A, the most common type of SCLC, the CD8-high group was associated with favorable PFS (*P* = 0.025) (Fig. S2A) and OS (*P* = 0.010) (Fig. S2C) in the chemoimmunotherapy cohort. In contrast, such associations were not observed in the chemotherapy cohort (Fig. S2B and S2D). The addition of ICI had a favorable association with PFS (*P* = 0.031) (Fig. S3A) and favorable trend with OS (*P* = 0.40) (Fig. S3B) in the CD8-high group but not the CD8-low group (data not shown) for the patients with SCLC-A.

## Discussion

In this study, we investigated the relationships between the IHC subtype of SCLC, HLA-class I expression in the tumor or infiltration of CD8-positive CTLs into the tumor, and patient survival (PFS and OS) utilizing FFPE specimens from patients diagnosed with ED-SCLC and treated with chemotherapy and/or ICIs as initial therapy. The presence of CD8-positive cell infiltration (CD8-high) showed a significant favorable association with both PFS and OS in the chemoimmunotherapy cohort. Previous studies have also found that infiltration of CD8-expressing cells has been associated with a favorable prognosis in various types of malignancies with or without ICIs (Sun et al. 2019; Mariya et al. 2014; Shimizu et al. 2019; Elkoshi 2022). Our results revealed that the addition of an ICI confers a better prognosis when SCLC cells have infiltration of CD8-expressing cells. Since we utilized bronchoscopically biopsied specimens to confirm the pathologic diagnosis before the initial therapeutic intervention, the total tumor size in each sample slide was less than 5 mm^2^. Notably, the prediction of prognosis was aided by the analysis of a small fraction of samples obtained from heterogeneous tumors and CD8 immunostaining, which is used in daily diagnostic practice, even without antibodies, for determining the IHC subtype of SCLC.

On the other hand, HLA class I expression in tumor cells, a putative prerequisite for CTL-mediated cancer immunotherapy, was not associated with prolonged PFS and OS, as assessed by multivariate analysis (Kubo et al. 2021). Since the occurrence of SCLC is highly associated with numerous mutagens contained in tobacco cigarettes, SCLC is considered to be a malignancy with a high mutation burden (Park et al. 2019). Nevertheless, SCLC has long been regarded as a low-HLA class I malignancy; therefore, ICIs are less effective (Funa et al. 1986; Doyle et al. 1985). Unexpectedly, our observations revealed that 38.7% (29/75) of the SCLC cases showed diffusely positive expression of HLA class I molecules. The expression of HLA class I molecules in SCLC has not been studied extensively since the publication of a report examining cell lines and a small number of clinical specimens more than 30 years ago and may warrant re-examination with a larger number of clinical samples that are properly subclassified based on the expression of transcription-related molecules.

Subgroup analysis based on the expression of transcription-related molecules in the IMpower133 trial, which showed that chemoimmunotherapy promoted OS in patients with SCLC, showed that patients with SCLC-I, classified according to gene expression, tended to benefit from combined ICI and chemotherapy treatments compared to patients with other subtypes (Gay et al. 2021). In our IHC investigation, SCLC-I was also associated with favorable PFS and OS in the chemoimmunotherapy cohort. For comparison between cohorts with and without ICI, the combination of ICI significantly enhanced the prognosis of SCLC-I. This is partly consistent with a previous report (Qi et al. 2022). Considering the fact that the majority of SCLC-I patients were classified as CD8-high, it can be inferred that SCLC-I possesses an immune microenvironment readily infiltrated by CD8-positive cells. On the other hand, the addition of ICIs to chemotherapy conferred a prolonged PFS in SCLC-A type only in the CD8-high group. SCLC-A is characterized by high expression of DLL3, which is regulated by ASCL1. DLL3 is considered to inhibit T cell trafficking and infiltration, thereby resulting in the so-called cold tumor microenvironment (Shirasawa et al. 2023). However, our SCLC-A cases consisted of tumors with both high and low CD8 expression, indicating the presence of an unidentified factor influencing the infiltration of CD8-positive cells. Notably, our study relied solely on IHC, a method feasible within a clinical setting, rather than gene expression analysis of transcription-related molecules. (Qi et al. 2022; Ding et al. 2022).

In the present study, poor PS was associated with unfavorable prognosis, which is consistent with a previous report (Morimoto et al. 2022). In patients with malignancy, poor PS is associated with cancer cachexia and sarcopenia that potentially underlie poor response to therapy (Shijubou et al. 2022).

Our study has some limitations. First, it was based on retrospective observations from a limited number of patients. Therefore, an appropriately designed prospective study with a larger sample size is required to verify our results, which should also consider other factors potentially associated with PFS or OS. Secondly, some patients with short observation periods were included, thus studies with long-term observations are required. Third, the available histopathology specimens were only of a very small size. Since all the specimens were biopsied to confirm the histopathological analysis, we could not obtain enough samples to analyze the expression of other relevant cancer immunological molecules, such as PD-L1 or CD4. The opposite effect, however, was that we encountered less ambiguity in the classification determination due to the heterogeneity exhibited by tumor cells. For accurate standardized assessment overcoming the tumor heterogeneity, further study with larger specimens is warranted. The assistance by artificial intelligence would contribute the standardized subtype classification (Huang et al. 2022).

In conclusion, our findings demonstrate that the infiltration of CD8-positive cells into the tumor, along with the IHC subtype of SCLC, serve as promising prognostic factors for patients with ED-SCLC who receive chemoimmunotherapy as first-line treatment. The addition of an ICI could improve prognosis of ED-SCLC with CD8-positive cell infiltration. Furthermore, upon categorizing SCLC into IHC subtypes, significant survival improvement was specifically noted in SCLC-I, despite the limited number of cases in this subtype. Additionally, the prognosis of SCLC-A, the predominant subtype of SCLC, was also enhanced when combined with an ICI only in cases where the tumor was infiltrated with CD8-positive cells. On the other hand, it remains inconclusive whether the infiltration of CD8-positive cells is also correlated with an improved prognosis by chemoimmunotherapy in SCLC-N and SCLC-P. We would like to emphasize that the presented results are based on IHC, a procedure that can be performed in daily clinical practice. It is suggested that the incorporation of the assessment of SCLC subtypes and CD8 cell infiltration into the SCLC treatment protocol involving ICI could offer substantial benefits to patients with SCLC and potentially lead to favorable medical economics. All that is necessary for the practical implementation of our findings in the routine clinical practice are acquiring and employing three antibodies targeting ASCL1, NEUROD1, and POU2F3, in addition to employing CD8 staining—a well-established technique in lymphoma diagnosis. Because this was a study with a limited sample size, further investigations with a larger population are required. Nevertheless, we believe that this study provides a valuable histopathological basis for future functional investigations of SCLC.

### Supplementary Information

Below is the link to the electronic supplementary material.

Supplementary file1 (DOCX 121 KB)

## Data Availability

The datasets used and/or analyzed during the current study are available from the corresponding author on reasonable request.

## Abbreviations

ASCL1: Achaete-scute homolog 1
CR: Complete response
CTL: Cytotoxic T lymphocyte
ECOG-PS: Eastern Cooperative Oncology Group performance status
ED-SCLC: Extensive-disease small-cell lung cancer
FFPE: Formalin-fixed paraffin-embedded
ICI: Immune checkpoint inhibitor
IHC: Immunohistochemistry
irAE: Immune-related adverse event
NEUROD1: Neurogenic differentiation factor 1
OS: Overall survival
PD: Progressive disease
PFS: Progression-free survival
POU2F3: POU class 2 homeobox 3
PR: Partial response
SD: Stable disease
YAP1: Yes-associated protein 1
