# Supplementary material for: Prognostic significance of immunohistochemical classification utilizing biopsy specimens in patients with extensive-disease small-cell lung cancer treated with first-line chemotherapy and immune checkpoint inhibitors
Source: J Cancer Res Clin Oncol. 2024 Mar 14;150(3):125. doi: 10.1007/s00432-024-05652-2 (PMC10940450; doi:10.1007/s00432-024-05652-2)
Supplement: Supplementary file 1 — Supplementary file1 (DOCX 121 KB) [file 432_2024_5652_MOESM1_ESM.docx]

**Prognostic significance of immunohistochemical classification utilizing biopsy specimens in patients with extensive-disease small-cell lung cancer treated with first-line chemotherapy and immune checkpoint inhibitors**

Naoki Shijubou^1,2^*, Toshiyuki Sumi^2.3^, Terufumi Kubo^1^*, Kenta Sasaki^1^, Tomohide Tsukahara^1^, Takayuki Kanaseki^1^, Kenji Murata^1^, Yoshiko Keira^4^, Kotomi Terai^4^, Tatsuru Ikeda^4^, Yuichi Yamada^3^, Hirofumi Chiba^2^, Yoshihiko Hirohashi^1^* and Toshihiko Torigoe^1^

^1^Department of Pathology, Sapporo Medical University School of Medicine, Sapporo, Japan.

^2^Department of Respiratory Medicine and Allergology, Sapporo Medical University School of Medicine, Sapporo, Hokkaido, Japan

^3^Department of Respiratory Medicine, Hakodate Goryokaku Hospital, Hakodate, Hokkaido, Japan

^4^Department of Pathology, Hakodate Goryokaku Hospital, Hakodate, Hokkaido, Japan

***Corresponding authors**:

Naoki Shijubou, Terufumi Kubo, and Yoshihiko Hirohashi

Department of Pathology, Sapporo Medical University School of Medicine, Sapporo 060-8556, Japan

Phone: +81-11-611-2111 (ext. 26910)

Fax: +81-11-643-2310

E-mails: shjibou.1229@gmail.com (N.S.), kuboteru@sapmed.ac.jp (T.K.), and hirohash@sapmed.ac.jp (Y.H.)

**Supplementary Tables**

**Table S1** Univariate and multivariate analysis of predictors of PFS in the chemotherapy cohort

| **Parameters** | **Univariate analysis**  **HR (95% CI)** | **P-value** | **Multivariate analysis**  **HR (95% CI)** | **P-value** |
| --- | --- | --- | --- | --- |
| Age ≥ 70 years | 0.87 (0.44–1.74) | 0.70 |  |  |
| Sex, female | 0.68 (0.28–1.68) | 0.41 |  |  |
| ECOG-PS ≥ 1 | 2.68 (1.26–5.69) | 0.010 | 2.44(1.14–5.22) | 0.022 |
| HLA I high | 0.99 (0.49–1.97) | 0.97 |  |  |
| CD8-high | 1.25 (0.63–2.45) | 0.52 |  |  |
| Subtype SCLC-N  (vs SCLC-I) | 0.53  (0.10–2.92) | 0.46 |  |  |
| Subtype SCLC-P  (vs SCLC-I) | 0.90  (0.57–1.43) | 0.66 |  |  |
| Subtype SCLC-A  (vs SCLC-I) | 0.69  (0.40–1.21) | 0.20 |  |  |
| C-Stage ≥ IVB | 2.08 (0.88–4.92) | 0.16 | 1.71 (0.72–4.05) | 0.22 |

CI: confidence interval; HR: hazard ratio.

**Table S2** Univariate and multivariate analysis of predictors of OS in the chemotherapy cohort

| **Parameters** | **Univariate analysis**  **HR (95% CI)** | **P-value** |
| --- | --- | --- |
| Age ≥ 70 years | 1.19 (0.56–1.67) | 0.41 |
| Sex, female | 0.90 (0.31–2.62) | 0.85 |
| ECOG-PS ≥ 1 | 2.12 (0.98–4.60) | 0.058 |
| HLA I high | 1.25 (0.56–2.81) | 0.59 |
| CD8-high | 0.97 (0.47–2.04) | 0.95 |
| Subtype SCLC-N (vs SCLC-I) | 0.47 (0.040–5.68) | 0.56 |
| Subtype SCLC-P (vs SCLC-I) | 0.83 (0.38–1.81) | 0.64 |
| Subtype SCLC-A (vs SCLC-I) | 1.05 (0.57–1.93) | 0.88 |
| C-Stage ≥ IVB | 1.86 (0.78–4.42) | 0.16 |

CI: confidence interval; HR: hazard ratio.

**Supplementary** **Figures**

**
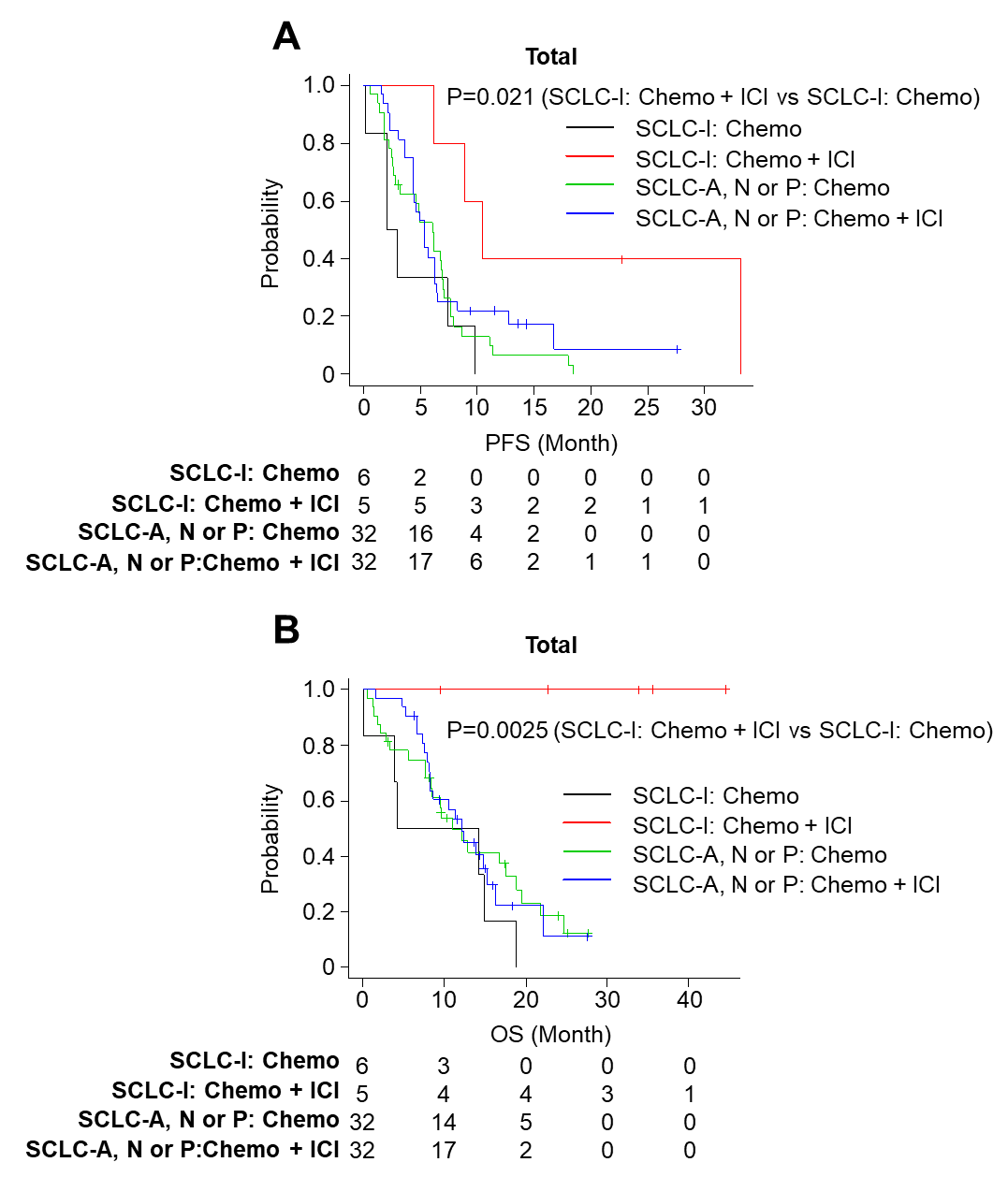
**

**Fig. S1** Kaplan–Meier estimates of PFS and OS in SCLC-I and the other subtype

(A) PFS of chemoimmunotherapy and chemotherapy cohorts in SCLC-I and the other subtype. (B) OS of the chemoimmunotherapy (chemo + ICI) and chemotherapy (chemo) cohorts in SCLC-I and the other subtype


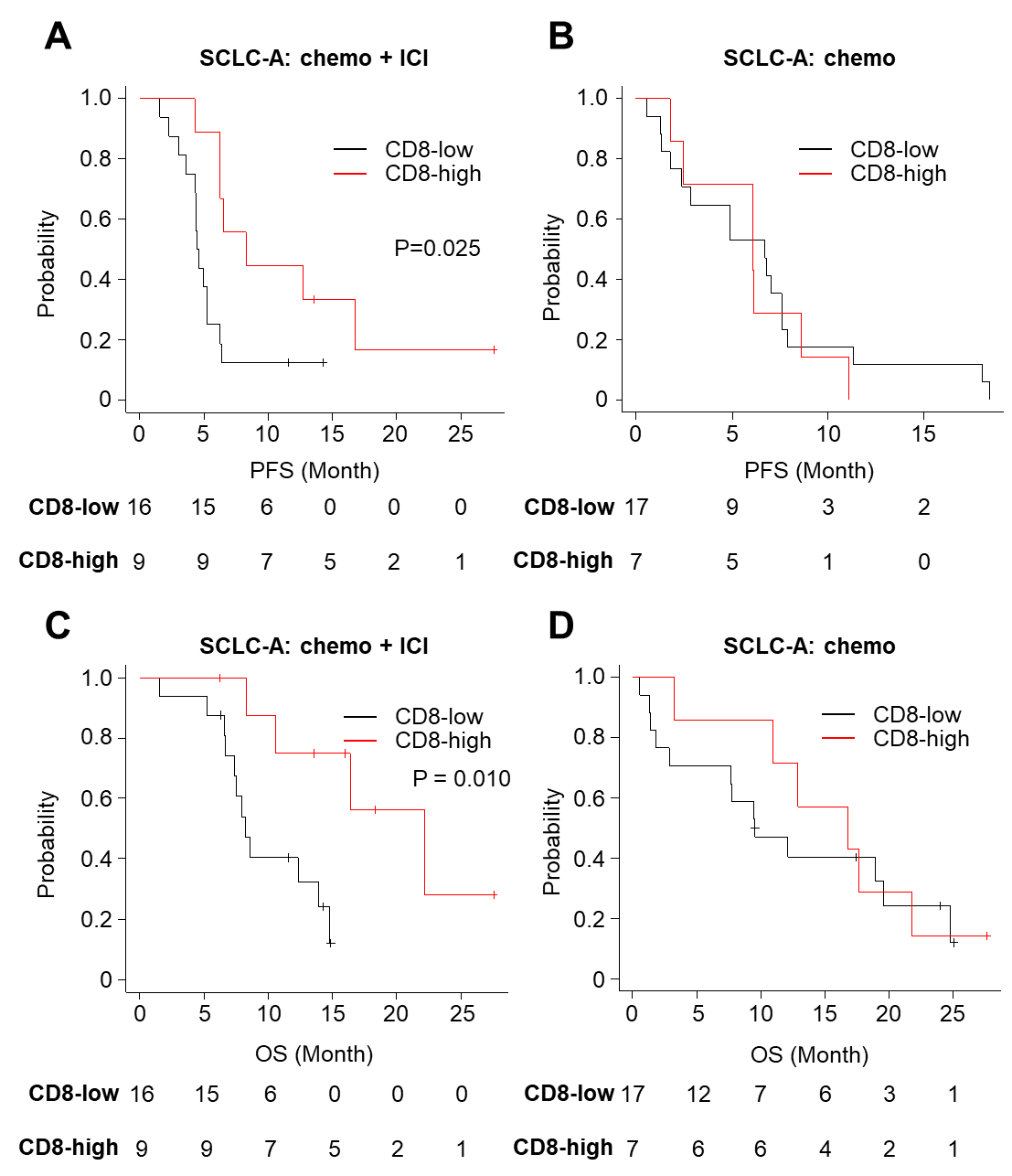


**Fig. S2** Kaplan–Meier estimates of PFS and OS in SCLC-A

PFS classification based on (A) the CD8-high or- low group in the chemoimmunotherapy cohort and (B) CD8-high or -low group in the chemotherapy cohort. OS classification based on (C) the CD8-high or- low group in the chemoimmunotherapy cohort and (D) the CD8-high or -low group in the chemotherapy cohort.

**
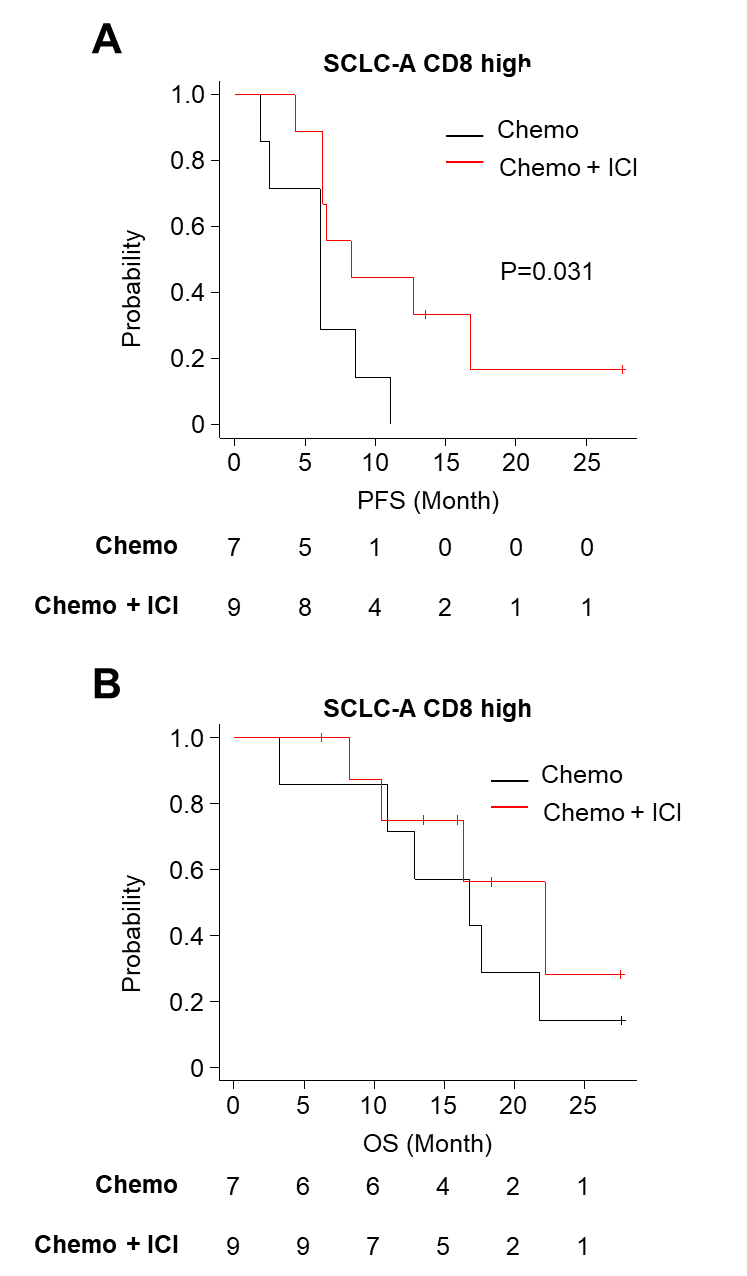
**

**Fig. S3** Kaplan–Meier estimates of PFS and OS in the CD8-high group of SCLC-A

(A) PFS of chemoimmunotherapy and chemotherapy cohorts in the CD8-high group of SCLC-A. (B) OS of the chemoimmunotherapy (chemo + ICI) and chemotherapy (chemo) cohorts in the CD8-high group of SCLC-A.
